# NAFLD Associates with Sarcopenia Defined by Muscle Mass and Slow Walking Speed: A Cross-Sectional Analysis from the Framingham Heart Study

**DOI:** 10.3390/jcm12247523

**Published:** 2023-12-06

**Authors:** Sarah Altajar, Na Wang, Max P. Rosenthaler, Joanne M. Murabito, Michelle T. Long

**Affiliations:** 1 Division of Gastroenterology and Hepatology, University of Miami Health System, Miami, FL 33136, USA; sarah.altajar@jhsmiami.org; 2 Biostatistics and Epidemiology Data Analytics Center, Boston University School of Public Health, Boston, MA 02118, USA; nwang10@bu.edu; 3 Department of Internal Medicine, Boston Medical Center, Boston University School of Medicine, Boston, MA 02118, USA; murabito@bu.edu; 4 Section of Gastroenterology, Boston Medical Center, Boston University School of Medicine, Boston, MA 02118, USA; mtlong@bu.edu

**Keywords:** muscle strength, muscle performance, NAFLD, functional status, obesity

## Abstract

Sarcopenia is associated with NAFLD. It is unknown if the association is explained by shared risk factors. Our study sought to investigate the association between liver fat and sarcopenia in our cohort. Liver fat was measured on CT between 2008 and 2011. We excluded heavy alcohol use and missing covariates. Muscle mass in a subset (n = 485) was measured by 24 h urinary creatinine. Physical function was defined by h strength and walking speed. Sarcopenia was defined as low muscle mass and/or low physical function. We created multivariable-adjusted regression models to evaluate cross-sectional associations between liver fat and low muscle mass, grip strength, and walking speed. The prevalence of hepatic steatosis was 30% (n = 1073; 58.1% women; mean age 65.8 ± 8.6 years). There was a significant positive association between liver fat and muscle mass in linear regression models. The association was not significant after adjusting for BMI. The odds of sarcopenia increased by 28% for each SD in liver fat (OR 1.28; 95% CI 1.02, 1.60) and persisted after accounting for confounders in multivariable-adjusted models (OR 1.30, 95% CI 1.02, 1.67). Further studies are needed to determine if there is a causal relationship between liver fat and sarcopenia and whether treatment of sarcopenia improves liver fat.

## 1. Introduction

Sarcopenia, which is characterized by the progressive loss of strength and skeletal muscle mass, is common among patients with many different diseases [1]. In particular, sarcopenia is increasingly recognized as an important disease phenotype in individuals with obesity [2,3,4]. Emerging data suggest an association between non-alcoholic fatty liver disease (NAFLD), an obesity-related condition, and sarcopenia [5]. Obesity and NAFLD, as closely related conditions, share many potential mechanisms that could worsen sarcopenia, including increased inflammation [6], hormonal derangements [2], altered myokines secreted by skeletal muscle (myostatin, adiponectin) [7,8], physical inactivity [9], and insulin resistance [10]. It is not known if the association between NAFLD and sarcopenia may be fully or partially explained by shared risk factors [11].

Prior studies on the association between NAFLD and sarcopenia are limited by a lack of objective measurements of liver fat and incomplete adjustments for confounding factors [3,12]. Additionally, many of the population-based studies were based in Asia, and thus, the results may be less generalizable to the US population [3,5,12,13]. A Korean population-based study that defined NAFLD by the fatty liver index observed an association between NAFLD and sarcopenia; however, the possibility of residual confounding remains since body mass index (BMI) and waist circumference are included in the fatty liver index definition of NAFLD and, therefore; could not be accounted for in adjusted models [5]. Multiple studies have explored the association between NAFLD and sarcopenia, defined by low muscle mass [3,5]. Recently, an international working group on sarcopenia recommended using both measures of low muscle mass and low muscle function, such as measures of strength or performance, for the diagnosis of sarcopenia [4]. However, most prior studies singularly focused on measures of muscle mass and did not consider functional measures of muscle strength or performance [3,5].

Thus, we aimed to investigate the cross-sectional association between computed tomography (CT)-defined NAFLD and the prevalence of sarcopenia as defined by muscle mass, muscle strength, and walking speed in a large, community-based cohort study.

## 2. Materials and Methods

Study sample: Participants were drawn from the Framingham Heart Study (FHS) Offspring Cohort who had previously undergone multi-detector computed tomography (CT) scan between 2008 and 2011 (n = 1632) [14]. Covariates and outcome measures were drawn from the nearest FHS clinical exam, the Offspring 8 examination (2005–2008), with the exception of muscle mass, which was only measured at the Offspring 9 examination (2011–2014). Participants were excluded if they met criteria for heavy alcohol use (>14 drinks/week for women and >21 drinks per week for men) (n = 552), had missing covariates (n = 6) or missing outcome variables (n = 1), which yielded a sample size of 1073 participants. Since measurements of muscle mass, muscle strength and physical performance were not available for all participants, we used different sample sizes for each analysis in order to include as many participants as possible. Muscle mass, derived from a 24 h urine collection, was available on 485 participants. Hand-grip strength was not available for one participant (final sample size n = 1072), and walking speed was missing from six participants (final sample size n = 1067). The study protocol was approved by the institutional review boards at Boston University School of Medicine and Massachusetts General Hospital, and all participants provided written informed consent.

Defining hepatic steatosis on computed tomography: The protocol for quantifying hepatic steatosis on CT scan has previously been described [15]. For the computed tomography study, participants were excluded if they weighed more than 160 kg, and only women > 40 years of age and men > 35 years of age had available data. Briefly, the participants underwent supine abdominal CT scanning of the abdomen, where twenty-five contiguous five mm slices were obtained. A radiographic phantom was present in each image. We averaged the liver attenuation in Hounsfield Units (HU) from three representative areas of the liver and divided the average liver attenuation by the attenuation of the phantom to define the liver-to-phantom ratio (LPR) [15]. Our primary exposure was defined as the continuous LPR (with a lower LPR indicating more liver fat). We defined hepatic steatosis by a binary LPR cut-off where LPR ≤ 0.33 was defined as the presence of hepatic steatosis [15].

Measurement of covariates: Smoking status and alcohol use were determined by clinician-administered questionnaires at the research exam visit. Participants were categorized as current smokers if they smoked one or more cigarettes within the last year. We recorded alcohol use in drinks per week or drinks per month. We measured blood glucose, high-density lipoprotein (HDL), total cholesterol, and triglycerides from fasting morning samples. Diabetes was defined as fasting blood glucose > 125 mg/dL or use of insulin or oral hypoglycemic medications. We categorized anti-hypertensive or lipid-lowering medication use based on the participant’s medication list. Trained research staff measured height, weight, and blood pressure (repeated once) at each visit. BMI was defined by weight in kg divided by height in meters squared (kg/m^2^). We defined hypertension by an average systolic blood pressure of ≥140 mm Hg or average diastolic blood pressure ≥ 90 mm Hg, or the use of anti-hypertensive medications.

Defining muscle mass and physical performance measures: The primary outcomes of interest were continuous measures of muscle mass and performance. We defined muscle mass by the 24 h urinary creatinine excretion, which is a reliable marker of total body muscle mass [16,17,18] and has been described previously in detail [19]. Briefly, participants were asked to collect urine for 24 h, starting with the post-morning void and completing after the morning void on day two. After completion, a 50 cc aliquot was removed and sent to Litholink (Chicago, IL, USA), where urinary creatinine was assessed using the photometric-kinetic alkaline picrate method. There were no significant exclusion criteria for 24 h urinary creatinine collection, and collection was offered to all participants during the period of collection. Ultimately, the group that had collections was slightly younger (69.7 vs. 71.2 years), less likely to use hypertension medication (54% vs. 60%), and less likely to smoke (5% vs. 9%), but otherwise similar to the general cohort. Measures of physical performance included hand-grip strength and walking speed. Hand-grip strength was measured by an adjustable hydraulic hand dynamometer (JAMAR, Sammons Preston, Inc., Bolingbrook, IL, USA) as described in a previous study [20]. We determined sex-specific medians and sex-adjusted measures of grip strength. Three trials were performed per hand with a three-second squeeze. We defined a participant’s grip strength by the highest of the six trials [20]. We defined walking speed as the fastest of two four-meter walks timed to the nearest 0.01 s by handheld stopwatch, during which participants were instructed to walk at their usual pace [20]. Secondary outcomes included the dichotomized outcome variables of low muscle mass, weak hand grip, or slow walking speed. Participants were considered to have low muscle mass, weak hand grip, or slow walking speed if their values were less than the sex-specific median for the study sample. We also considered an alternative definition of slow walking speed defined by walking speed < 1 m/s based on existing literature [21,22]. We defined sarcopenia as the combination of low muscle mass along with poor physical performance (low muscle mass and/or weak grip strength and low muscle mass and/or slow walking speed) [4].

### Statistical Analysis

We log-transformed muscle mass because the distribution was skewed. We performed multivariable-adjusted linear regression models to examine the association between liver fat and continuous measures of muscle mass, h strength, and walking speed. In the base model, we adjusted for age and sex. We included a multivariable model where, in addition to age and sex, we adjusted for vascular measurements (hypertension, systolic blood pressure, and diastolic blood pressure), diabetes, and lipid profile measurements (HDL, total cholesterol, triglycerides) or use of lipid-lowering medication. In a final model, we added adjustment for BMI to the multivariable model. These models were defined a priori, and no covariates were removed from the models based on the significance of association. We performed multivariable-adjusted logistic regression models to examine the association between liver fat and dichotomous measures of low muscle mass, weak grip strength, slow walking speed, and sarcopenia. We adjusted for the same covariates as with the linear regression models. We also tested for interactions with sex and obesity; however, since no interactions were observed, we did not stratify models based on these variables. Beta estimates and odds ratios (OR) were presented per standard deviation decrease in LPR (representing an increase in liver fat). All analyses were performed using SAS version 9.4 (SAS Institute, Cary, NC, USA). A *p*-value of <0.05 was considered statistically significant for all analyses.

## 3. Results

### 3.1. Study Sample Characteristics

The baseline characteristics of our study sample (n = 1073) stratified by hepatic steatosis status are shown in Table 1. The sample included 58.1% women, and the mean ± standard deviation (SD) age was 65.8 ± 8.6 years. Approximately 30% of the sample had CT-defined hepatic steatosis. Participants with hepatic steatosis were more frequently men and had a higher prevalence of diabetes, hypertension, high cholesterol, and low muscle mass compared to those without hepatic steatosis (*p* < 0.005 for all). Participant characteristics were similar in the sub-sample with muscle mass measurement.

### 3.2. Multivariable-Adjusted Linear Regression Models for the Association between Liver Fat and Muscle Mass, Grip Strength, and Walking Speed

In age- and sex-adjusted linear regression models (Table 2), we observed a significant positive association between liver fat and log-muscle mass (β 0.028; 95% confidence interval (CI) 0.01–0.046). The association between liver fat and log-muscle mass persisted in the multivariable-adjusted model (β 0.022; 95% CI 0.003–0.04; however, the association was attenuated and no longer significant after adding an adjustment for BMI to the multivariable model (β 0.36; 95% CI −0.01–0.27). We observed a negative association between liver fat and walking speed in age- and sex-adjusted models, but this association did not persist after adjusting for covariates. No association was observed between liver fat and h strength in any of the linear regression models. We did not observe interactions with sex or obesity.

### 3.3. Multivariable-Adjusted Logistic Regression Models for the Association between Liver Fat and with Low Muscle Mass, Weak Grip Strength, Slow Walking Speed, and Sarcopenia

In multivariable-adjusted logistic regression models, we observed a significant association between liver fat and slow walking speed (OR 1.19; 95% CI 1.05–1.36) in age- and sex-adjusted models; however, this association was no longer significant after multivariable adjustment (Table 3). We did not observe significant associations between liver fat and either low muscle mass or weak grip strength.

We also considered sarcopenia as the combined outcomes of low muscle mass and/or weak grip strength and low muscle mass and/or slow walking speed (defined by walking speed <median). The odds of sarcopenia (defined as low muscle mass and/or slow walking speed) increased by 28% for each standard deviation increase in liver fat (OR 1.28; 95% CI 1.02–1.60), and this association persisted after accounting for covariates (OR 1.34; 95% CI 1.05–1.71) and BMI (OR 1.30; 95% CI 1.02–1.67) (Table 3).

In a secondary analysis using an alternative definition for slow walking speed (<1 m/s), we did not observe any significant association between liver fat and sarcopenia defined by low muscle mass and/or slow walking speed. There was no significant association between liver fat and sarcopenia defined by low muscle mass and/or weak grip strength in our multivariable-adjusted models.

## 4. Discussion

### 4.1. Principle Findings

In our community-based cohort study, we had the opportunity to evaluate the association between objectively defined liver fat, muscle mass, and several measures of physical performance to examine the complicated relationship between NAFLD, obesity, and sarcopenia. When considering individual measures of muscle mass, grip strength, or walking speed, we did not observe any significant associations with liver fat after accounting for shared cardiometabolic risk factors and obesity. However, when we considered a definition of sarcopenia based on a combination of muscle mass and function, as measured by walking speed, we observed an association between more liver fat and sarcopenia that persisted even after accounting for cardiometabolic risk factors, including obesity. Taken together, our findings suggest that considerations of both muscle mass and function may be important when considering the association between NAFLD and sarcopenia.

### 4.2. In the Context of the Current Literature

Sarcopenia is associated with worse clinical outcomes, morbidity, and mortality in patients with liver disease [23,24]. Given the high prevalence of NAFLD, the relationship between sarcopenia and NAFLD has been of increasing interest. Several population-based studies have investigated the relationship between NAFLD and sarcopenia; however, few studies have considered both muscle mass and physical performance in the definition of sarcopenia [3,5,12,13,25]. In a small cross-sectional study based in Korea, CT-defined NAFLD was associated with sarcopenia (defined by dual-energy X-ray absorptiometry (DEXA)); however, this study did not account for obesity in the adjusted models [13]. In a larger cross-sectional study also based in Korea, NAFLD was defined by diagnostic scores, which include BMI, so it is difficult to interpret the association between score-defined NAFLD and DEXA-defined sarcopenia since those with a higher BMI were also more often characterized as NAFLD [5]. One prior study that considered both DEXA-defined muscle mass and measures of physical performance (hand-grip strength and walking speed) did not observe an association between NAFLD and sarcopenia. However, the sample size was small, and the combined effects of low muscle mass and low physical performance were not considered [12]. In our study, we examined the combined effect of low muscle mass and low physical performance to define sarcopenia, which may explain why we observed an association between liver fat and sarcopenia and the prior study did not. In other studies, the associations between NAFLD and sarcopenia did not include measures of physical performance [3,25].

A major difference between our study and previous studies on the association between NAFLD and sarcopenia was in how we defined sarcopenia. Our study is the first to use 24 h creatinine as an estimate for muscle mass as opposed to the imaging estimates used by other studies. Though less reproducible compared to DEXA-based estimates, 24 h creatinine has been shown to be a more sensitive and accurate marker of muscle mass [18]. DEXA and other imaging estimates of muscle mass may overestimate lean muscle mass since these methods may have difficulty differentiating between muscle edema, which increases with age, and lean muscle mass [18,26].

When considering sarcopenia, we observed associations with liver fat when we considered low muscle mass along with slow walking speed but not weak grip strength. Many consider walking speed, which declines with age and is influenced by neuromuscular, cardiorespiratory, and musculoskeletal systems, to be a summary indicator of frailty [27,28]. Though walking speed and hand-grip strength are both associated with cardiovascular mortality, the associations are more consistent for walking speed compared to hand-grip strength [29]. One prospective study of grip strength demonstrated a strong association between grip strength and all-cause and cardiovascular mortality but no association with incident diabetes [30]. Additional studies are needed to understand which physical performance measurements are most helpful in predicting poor outcomes for persons with liver disease.

### 4.3. Potential Mechanisms

NAFLD and sarcopenia are both strongly associated with obesity [2,31]. One proposed mechanism for the association of NAFLD and sarcopenia is that they are both disease manifestations of the consequences of obesity, namely insulin resistance, physical inactivity, and pro-inflammatory state [31,32,33,34,35,36]. However, several prior studies, in addition to the present investigation, have demonstrated a persistent association between NAFLD and sarcopenia despite adjusting for BMI, obesity, and insulin resistance. Mediators along the adipose tissue-muscle-liver axis may play a role in promoting NAFLD. In animal models, myostatin, a regulator of skeletal muscle mass, protects mice from liver fat and improves insulin sensitivity [8,37]. Additionally, with skeletal muscle atrophy, there may be alterations in total body energy expenditure since skeletal muscle mass contributes to both resting and maximum energy expenditure [21,38]. Lower energy expenditure may contribute to the development and progression of fatty liver disease, though additional studies are needed [39].

### 4.4. Implications

The growing insight into the association between sarcopenia and NAFLD may impact prognostication for patients, given that sarcopenia has been linked with morbidity and mortality in liver cirrhosis [40,41] and early-stage liver disease [24]. Furthermore, NAFLD-associated sarcopenia might represent a potential target for the prevention and treatment of NAFLD and/or NAFLD-related morbidity through proactive and early muscle strengthening [42,43]. Finally, establishing the mechanistic underpinnings of NAFLD and sarcopenia provides a possible target for the development of medical therapies to treat NAFLD. Potential targets of NAFLD-associated sarcopenia include myostatin antibodies, growth hormone, testosterone, and ammonia-lowering agents [44]. However, as of yet, these medical therapies are poorly studied and will require further investigation before becoming an option for managing NAFLD-associated sarcopenia.

### 4.5. Strengths and Limitations

The major strength of our study is the use of a well-characterized, community-based cohort in which data were obtained in a standardized manner. Important limitations are worth noting. First, our study is limited by its observational nature, and thus, we are unable to comment on causality. The Framingham Heart study cohort is largely white and of European ancestry, limiting generalizability to diverse populations. Additionally, CT imaging, which is insensitive to mild liver fat, may under-represent the true prevalence of NAFLD in the study population. CT imaging is also insensitive to hepatic fibrosis, and we did not have other non-invasive measures of hepatic fibrosis in our cohort. However, based on our prior work, the prevalence of advanced fibrosis or cirrhosis in our cohort is likely low and not a significant source of confounding [45]. Another limitation is that we did not have information on viral hepatitis status and medication usage, which may have contributed to misclassification and biased our results towards the null. However, the effect of misclassification should be minimal since the prevalence of viral hepatitis and usage of offending medications (such as steroids, amiodarone, and tamoxifen) will be relatively low in our cohort. Our sample was not enriched for participants with weak grip strength or slow walking speed, and we had a limited number of participants with clinically significant weakness [45], which may have biased our study towards the null. An additional limitation is that physical activity, which is an important potential confounding variable with liver fat and sarcopenia, is unmeasured in our population. Finally, we used 24 h urinary creatinine excretion as a measure for muscle mass, and this measure can be subject to variability compared to imaging-based estimations of muscle mass as it depends on accurate collection of urine, a stable diet, and renal function [46]. We do not have available measurements of muscle mass in our dataset to be able to validate the urinary creatinine-based estimates. We did not use the European Working Group on Sarcopenia in Older People (EWGSOP2) criteria to define sarcopenia because of limitations in what was available in our cohort study, which may have impacted our measurement of muscle mass. These guidelines were not published when we measured muscle mass by 24 h urine creatinine in our cohort. Additionally, in persons with chronic disease, urine creatinine may not have the same level of reliability as a measure of muscle mass [47] and this limitation may have led to additional confounding in our study.

## 5. Conclusions

In our community-based cohort study, we observed significant associations between liver fat and sarcopenia as defined by low muscle mass and/or slow walking speed. Further studies are needed to determine the causal association between liver fat and sarcopenia and whether treatment of sarcopenia improves liver fat.

## Figures and Tables

**Table 1:** Participant characteristics.

Clinical Characteristic	Hepatic Steatosis ^a^ (n = 321)	No Hepatic Steatosis (n = 751)	Total Sample (n = 1073)
Age (years)	65.6 ± 8.3	65.8 ± 8.7	65.8 ± 8.6
Women, N (%)	158 (49.2)	465 (61.8)	623 (58.1)
Body mass index (kg/m^2^)	30.8 ± 5.3	27.3 ± 4.9	28.4 ± 5.2
Alcohol (drinks/week)	4.9 ± 5.7	4.4 ± 5.1	4.5 ± 5.3
Physical activity index	35.0 ± 5.3	35.8 ± 5.3	35.6 ± 5.3
Current Smoking, N (%)	13 (4.0)	46 (6.1)	59 (5.5)
Diabetes, N (%)	69 (21.5)	68 (9.0)	137 (12.8)
Anti-hypertensive treatment, N (%)	177 (55.3)	291 (38.7)	468 (43.7)
Hypertension, N (%)	203 (63.2)	362 (48.1)	565 (52.7)
Systolic blood pressure (mm Hg)	129 ± 15	126 ± 17	127 ± 16
Diastolic blood pressure (mm Hg)	74 ± 10	73 ± 10	73 ± 10
Total Cholesterol (mg/dL)	181 ± 34	189 ± 37	187 ± 37
HDL Cholesterol (mg/dL)	51 ± 14	59 ± 17	57 ± 17
Triglycerides (mg/dL)	138.2 ± 76.5	106.6 ± 54.2	116.1 ± 63.3
Lipid-lowering medication, N (%)	162 (50.5)	302 (40.2)	464 (43.3)
ALT (µ/L)	27.9 ± 14.6	23.8 ± 10.8	25.0 ± 12.2
H strength ^b^ (kg)	33.0 ± 12.0	30.7 ± 11.1	33.0 ± 11.4
Weak grip strength, N (%)	153/320 (47.8)	352/751 (46.8)	505/1072 (47.1)
Walking speed ^c^ (m/s)	1.14 ± 0.27	1.16 ± 0.25	1.15 ± 0.26
Slow walking speed, N (%)	167/319 (52.5)	359/748 (47.9)	526/1067 (49.3)
Slow walking speed < 1 m/s, N (%)	92/319 (28.9)	203/748 (27.1)	295/1067 (27.6)
Muscle mass ^d^ (mg/day)	1393 (1080, 1809)	1206 (996, 1581)	1242 (1023, 1642)
Low muscle mass, N (%)	51/129 (39.5)	191/356 (53.7)	242/485 (49.9)

Continuous variables expressed as mean ± sd, categorical variables as n (%), except as noted. Abbreviations: HDL—high-density lipoprotein; ALT—alanine aminotransferase. ^a^ As defined by liver phantom ratio ≤ 0.33. ^b^ Sample size with hepatic steatosis n = 320, no hepatic steatosis n = 751, total n = 1072. ^c^ Sample size with hepatic steatosis n = 319, no hepatic steatosis n = 748, total n = 1067. ^d^ As defined by 24 h urinary creatinine and expressed in median (Interquartile range); sample size with hepatic steatosis n = 129, no hepatic steatosis n = 356, total n = 485.

**Table 2:** Multivariable-adjusted linear regression models examining the cross-sectional association between continuous liver fat and muscle mass ^a^, hand-grip strength, and walking speed.

	Log-Muscle Mass ^a^		Hand Grip Strength		Walking Speed	
Sample Size	N = 485		N = 1072		N = 1067	
Model	ß [95% CI]	*p* Value	ß [95% CI]	*p* Value	ß [95% CI]	*p* Value
Age- and sex-adjusted	0.028 (0.010, 0.046)	0.002	−0.115 (−0.519, 0.289)	0.58	−0.019 (−0.033, −0.005)	0.007
MV ^b^-adjusted	0.022 (0.003, 0.040)	0.02	−0.016 (−0.444, 0.413)	0.94	−0.011 (−0.026, 0.004)	0.16
MV ^b^ + BMI-adjusted	0.009 (-0.010, 0.027)	0.36	0.006 (−0.440, 0.452)	0.98	−0.001 (−0.017, 0.014)	0.85

Abbreviations: BMI—body mass index; CI—confidence interval; MV— multivariable. ^a^ As measured by 24 h urinary creatinine excretion. ^b^ MV model adjusts for age, sex, systolic blood pressure, diastolic blood pressure, hypertension, smoking, total cholesterol, high-density lipoprotein, and triglycerides.

**Table 3:** Multivariable-adjusted logistic regression models examining the cross-sectional relationship between continuous liver fat ^a^ and low muscle mass ^b^, weak grip strength ^c^, low gait speed ^d^, and sarcopenia.

	Low Muscle Mass ^b^		Weak Grip Strength ^c^		Slow Walking Speed ^d^		Sarcopenia: Low Muscle Mass ^b^ and/or Weak Grip Strength ^c^		Sarcopenia: Low Muscle Mass ^b^ and/or Slow Walking Speed ^d^	
Sample Size	N = 242		N = 505		N = 526		N = 333		N = 342	
Model	OR (95% CI)	*p* Value	OR (95% CI)	*p* Value	OR (95% CI)	*p* Value	OR (95% CI)	*p* Value	OR (95% CI)	*p* Value
Age- and sex-adjusted	0.79 (0.59, 1.06)	0.12	1.09 (0.96, 1.24)	0.19	1.19 (1.05, 1.36)	0.008	0.97 (0.79, 1.20)	0.79	1.28 (1.02, 1.60)	0.03
MV ^e^-adjusted	0.85 (0.62, 1.16)	0.31	1.05 (0.91, 1.21)	0.52	1.12 (0.98, 1.29)	0.10	0.97 (0.78, 1.22)	0.81	1.34 (1.05, 1.71)	0.02
MV ^e^ + BMI-adjusted	0.96 (0.70, 1.33)	0.81	1.05 (0.91, 1.22)	0.50	1.05 (0.91, 1.21)	0.53	1.05 (0.83, 1.33)	0.69	1.30 (1.02, 1.67)	0.04

Abbreviations: OR—odds ratio; MV—multivariable; CI—confidence interval; BMI—body mass index. ^a^ Defined as a continuous liver–phantom ratio. ^b^ As defined by <median 24 h urinary creatinine excretion. ^c^ As defined by <median hand-grip strength. ^d^ As defined by <median walking speed. ^e^ MV model adjusts for age, sex, systolic blood pressure, diastolic blood pressure, hypertension, smoking, total cholesterol, high-density lipoprotein, and triglycerides.

## Data Availability

Data will be available upon request.

## Abbreviations

FHS	Framingham Heart Study
SD	Standard Deviation
CI	Confidence Interval
OR	Odds Ratio
NAFLD	Non-Alcoholic Fatty Liver Disease
CT	Computed Tomography
HU	Hounsfield Unit
LPR	Liver to Phantom Ratio
HDL	High-Density Lipoprotein
BMI	Body Mass Index
